# HDV infection rates in northern Vietnam

**DOI:** 10.1038/s41598-018-26446-w

**Published:** 2018-05-23

**Authors:** Mai Thanh Binh, Nghiem Xuan Hoan, Hoang Van Tong, Dao Phuong Giang, Bui Tien Sy, Nguyen Linh Toan, Le Huu Song, Mai Hong Bang, Heiner Wedemeyer, Christian G. Meyer, Peter G. Kremsner, C.-Thomas Bock, Thirumalaisamy P. Velavan

**Affiliations:** 1 Institute of Tropical Medicine, University of Tübingen, Tübingen, Germany; 2 Vietnamese-German Center for Excellence in Medical Research, Hanoi, Vietnam; 3 108 Military Central Hospital, Hanoi, Vietnam; 4 Vietnamese Military Medical University, Hanoi, Vietnam; 5 Department of Infectious Diseases, Robert Koch Institute, Berlin, Germany; 6 Department. for Gastroenterology and Hepatology Medical Center, University Hospital Essen, Essen, Germany; 7 Faculty of Medicine, Duy Tan University, Da Nang, Vietnam

## Abstract

Hepatitis D caused by the hepatitis delta virus (HDV) is a serious health problem in many regions of the world. A total of 546 HBV-infected patients were enrolled from 2013 to 2015 and classified clinically into the subgroups of chronic hepatitis B (CHB, n = 191), liver cirrhosis (LC, n = 147) and hepatocellular carcinoma (HCC, n = 208). The patients were screened for HDV-RNA by nested PCR assays. HDV genotypes were assessed by direct sequencing, followed by phylogenetic analysis. HDV-RNA was identified in 13% (71/546) of HBV-infected patients. The highest HDV prevalence was found in the LC group (19.7%), followed by the HCC (12%) and CHB (8.9%) groups (*P* = 0.017). HDV/HBV coinfections were significantly associated with a rather unfavourable clinical outcome, in particular with LC development compared to HBV monoinfection. Phylogenetic analyses indicated that the genotype HDV1 was, with a prevalence of 91%, by far the most common genotype in Vietnam, followed by HDV2 with 9%. Other HDV genotypes were not observed. In accordance with previous data obtained a decade ago, our results confirm a continuing high prevalence of HDV infection in hepatitis B patients in northern Vietnam with the HDV1 genotype still being the predominant genotype. HDV nucleic acid testing to minimize the associated risk should be considered.

## Introduction

Hepatitis delta virus (HDV), firstly identified in 1977^1^, is a defective virus which uses hepatitis B virus (HBV) envelope proteins for successful infection of hepatocytes^2^. The HDV virion is composed of an outer coat containing HBV envelope proteins and host lipids surrounding an inner nucleocapsid that consists of small and large hepatitis delta antigens (HDAg) and a single-stranded circular RNA of 1679 nucleotides^3^. Both HBV and HDV use the Na+-taurocholate cotransporting polypeptide (NTCP) bile transporter to gain entry to the hepatocytes. HBV infections produce envelope proteins to assemble new HBV particles. HDV utilizes these envelope proteins for its own assembly. Given that HBV and HDV share the same envelope proteins, they enter hepatocytes by a similar mechanism. The viruses are transmitted to healthy individuals by concurrent infection either with HBV (coinfection) or by superinfection (superimposed on chronic hepatitis B). The HDV superinfection progress to an acute infection, leading to severe liver damage than HBV monoinfection.

HDV/HBV coinfections are a global health problem affecting 15–20 million people worldwide^4,5^. Although HDV infection usually is associated with an increased risk of liver cirrhosis (LC) and hepatocellular carcinoma (HCC)^3^, it is frequently underdiagnosed due to a lack of awareness and the unavailability of appropriate diagnostic tools in hospitals of developing countries. Moreover, HDV infection is not routinely tested in clinical practice in many of these countries and treatment of hepatitis D infections is challenging and largely ineffective^6,7^.

Currently, eight HDV genotypes (HDV1–8) had been recognized with distinct geographic distributions and associated clinical features^8–10^. The HDV1 genotype occurs worldwide and is associated with both severe and mild clinical forms of viral hepatitis^3^. The HDV2 genotype is mainly found in East Asia and causes mild disease^11^. HDV3 is predominantly found in South America^12^, and HDV4 preferentially occurs in Japan and Taiwan^13^. Genotypes HDV5-8 were identified in indigenous African ethnic groups, however, these genotypes, their prevalence and clinical relevance are less well characterized^3,9,11^.

The HDV prevalence in HBV infected patients is commonly described as percentage of anti-HDAg positive individuals in HBsAg-positive patients. Prevalences vary considerably across geographical regions. While HDV infections are rather rare in Europe due to largely effective HBV vaccination programs and screening of blood products, hepatitis D remains of concern with highest rates in low-income HBV-endemic countries with insufficient HBV vaccination coverage^14^. For example, high HDV seroprevalences were reported in Pakistan where vaccination campaigns are difficult to conduct (35.2%)^15^, Mongolia (67%)^16^, Gabon (15.6% to 70.6%)^17^. Recently, the HDV prevalence in sub-Saharan African countries was estimated to be 1.3% to 50%^18^.

In Vietnam, HBV infection rates range from 10% to 15% in the general population^19^, suggesting a high prevalence of HDV infection in HBsAg positive patients. Few studies only have assessed the epidemiological and clinical importance of HDV infection between 2000 and 2015 in Vietnam, indicating different results in terms of HDV infection prevalences across regions. In particular, the distribution of HDV genotypes differed between northern and southern Vietnam^20–23^. A high predominance of 90% of the HDV1 genotype in northern Vietnam was shown in HBV-infected patients recruited between 2000 and 2003^23^. In contrast, a recent study has reported that HDV2 was the predominant genotype (80%) in southern Vietnam^22^. However, both studies were conducted with small sample sizes of HBV patients only (n < 300). In the present study, a larger group of HBV-infected patients was involved in order to assess the prevalence of HDV infection and possible changes in genotype distributions over the last decade in the northern Vietnam. In addition, we assessed effects of HDV/HBV coinfections on the clinical staging of HBV-related liver diseases.

## Results

### Baseline characteristics of chronic hepatitis B patients

The demographic characteristics of the 546 HBV-infected study participants are summarized in Table 1. Of the 546 patients, 465 (85%) were male and 81 (15%) were female. In our study cohort, the mean age of patients was 53 (12–86 years). Eight individuals were at early adolescence (12–18 years old). Among those eight infected individuals, six were experiencing CHB (≤18 years) and one each were with LC and HCC (≤15 years). The HBV transmission was documented from mother to child in all those six patients among the infected eight. All eight patients were not vaccinated against HBV. As expected, patients in the CHB group were younger than those in LC and HCC groups (37 vs. 57 and 60 years, respectively). Albumin and prothrombin levels as well as platelet counts were significantly lower in the LC group compared to the other groups (*P* < 0.001) and direct and indirect bilirubin levels were higher in the LC compared to the CHB and HCC groups (*P* < 0.0001). Liver enzyme levels (AST, ALT) were significantly higher in the CHB group compared to the other groups (*P* = 0.0045 and 0.0048, respectively). AFP levels were significantly higher among HCC patients compared to the subgroups of CHB and LC patients (*P* < 0.0001).

**Table 1:** Clinical characteristics of 546 patients with chronic hepatitis B.

Characteristics	Total (n = 546)	CHB (n = 191)	LC (n = 147)	HCC (n = 208)	*P* value
Age (years)	53 [12–86]	37 [12–73]	57 [15–86]	60 [15–81]	<0.0001^#^
Male (%)	85.1	77	85	92.8	<0.0001^#^
**Child-Pugh classification**					
Child A		NA	60/132	153/208	
Child B		NA	61/132	47/208	
Child C		NA	11/132	8/208	
Missing		NA	15	0	
**Clinical parameters**					
AST (IU/L)	62.5 [14–6206]	47 [14–6206]	78 [18–1221]	58 [17–670]	0.0045^‡^
ALT (IU/L)	54 [8–3390]	60 [8–3390]	55 [8–1426]	48 [11–934]	0.0048^‡^
Total bilirubin (µmol/L)	18 [4.1–571]	16.3 [5.5–551]	31.65 [4.1–593]	17 [6–282]	<0.0001^‡^
Direct bilirubin (µmol/L)	6 [0.4–349]	5.9 [1–349]	11.5 [0.4–350.22]	5.5 [0.4–189.39]	<0.0001^‡^
albumin (g/L)	39 [9.8–49]	42 [9.8–48]	31 [15–47]	38 [21–49]	<0.0001^‡^
Prothrombin (% of standard)	85 [13–269]	92.5 [17–267]	60 [13–120]	83.5 [19.6–269]	<0.0001^‡^
PLT (×10^3^/ml)	174 [3.7–426]	210 [65–416]	89 [3.7–325]	166 [42–426]	<0.0001^‡^
HBV DNA (log_10_ copies/ml)	7.0 [2–10.3]	5.4 [2–10.3]	4.5 [2–9]	4.6 × 10^3^ [2–9.2]	0.019^‡^
AFP (IU/L)	8.4 [1.06–400]	3 [1.06–400]	7.9 [1.18–400]	133 [1.38–400]	<0.0001^‡^

CHB, chronic hepatitis B; LC, liver cirrhosis; HCC, hepatocellular carcinoma; PLT, platelets. AST and ALT, aspartate and alanine amino transferase; AFP, alpha-fetoprotein; IU, international unit. Values given are medians and ranges or percentile where appropriate. ^(‡)^ Kruskal-Wallis test. ^(#)^ Chi-square test.

### Prevalence of HDV infection in patients with HBV-related liver diseases

HDV-RNA was detected in 71 (13%) among the 546 HBV-infected. Of the 71 HDV-positive patients, 66/71 (93%) were male and 5/71 (7%) were female (*P* < 0.05). The prevalence of HDV infection among clinical groups is presented in Table 2. HDV-RNA was observed more frequently among LC (20%; 29/147) and HCC patients (12%; 25/208) compared to CHB patients (9% or 17/191) (*P* = 0.017). When LC patients were stratified according to child-pugh scores, HDV infections were significantly higher in patients with Child B or C compared to Child A score (*P* = 0.047), however such a significant trend could not be observed when stratified among HCC patients. These results suggest HDV infection as a significant risk factor for the progression of liver disease, esp. for liver cirrhosis.

**Table 2:** Association of HDV infection with liver disease progression.

Groups	HDV/HBV coinfection		HBV monoinfection		p value
**Clinical groups**	**n/total**	**(%)**	**n/total**	**(%)**	0.017
CHB	17/191	8.9	174/191	91.1	
LC	29/147	19.7	118/147	80.3	
HCC	25/208	12	183/208	88	
**Child-Pugh score**					
Child A	25/213	11.7	188/213	88.3	0.047
Child B	24/108	22.2	84/108	77.8	
Child C	3/19	15.8	16/19	84.2	
**BCLC staging**					
BCLC A	5/66	7.6	61/66	92.4	NS
BCLC B	13/76	17	63/76	83	
BCLC C	3/37	8	34/37	92	
BCLC D	1/10	10	9/10	90	

CHB, chronic hepatitis B; LC, liver cirrhosis; HCC, hepatocellular carcinoma; BCLC: Barcelona Clinic Liver Cancer; *P* values calculated by Chi-square test.

### HDV/HBV coinfection and biochemical parameters

**Figure 1:**
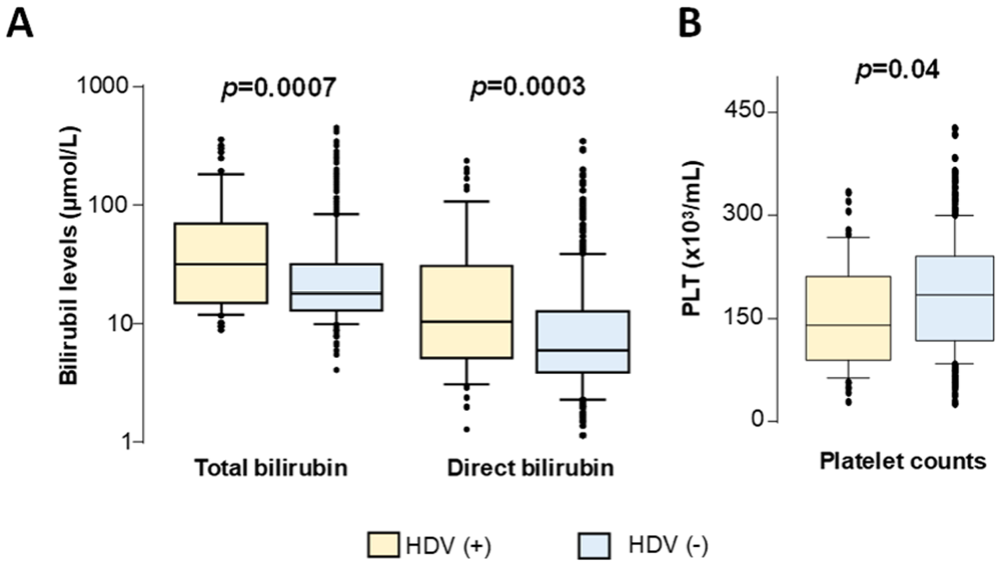
Association of HDV infection with subclinical parameters. Bilirubin levels (**A**) and Platelet counts (**B**) in HBV infected patients with HDV and without HDV coinfection. Other parameters (liver enzymes: AST and ALT, HBV DNA loads, albumin, prothrombin, AFP) that did not reach the statistical significance were not presented in the figure. Box-plots illustrate medians with 25 and 75 percentiles with whiskers to 10 and 90 percentiles. *P* values were calculated by Mann-Whitney-Wilcoxon test.

In order to analyze the influence of HDV infection on the clinical outcome in HBV-infected patients, we compared HBV-DNA loads, levels of liver enzymes as well as those of bilirubin, albumin, prothrombin and platelet counts between HBV patients with and without patent HDV infection. HBV-DNA levels were lower in HDV/HBV coinfected patients; however, the difference was not significant. Platelet counts were lower in coinfected patients compared to HBV monoinfected patients (*P* = 0.04). Both total and direct bilirubin levels were higher in HDV-infected patients than in those without HDV infection (*P* = 0.0007 and 0.0003, respectively) (Fig. 1). Comparisons of other laboratory parameters between HDV-positive and HDV-negative patients were not statistically significant (data not shown).

**Figure 2:**
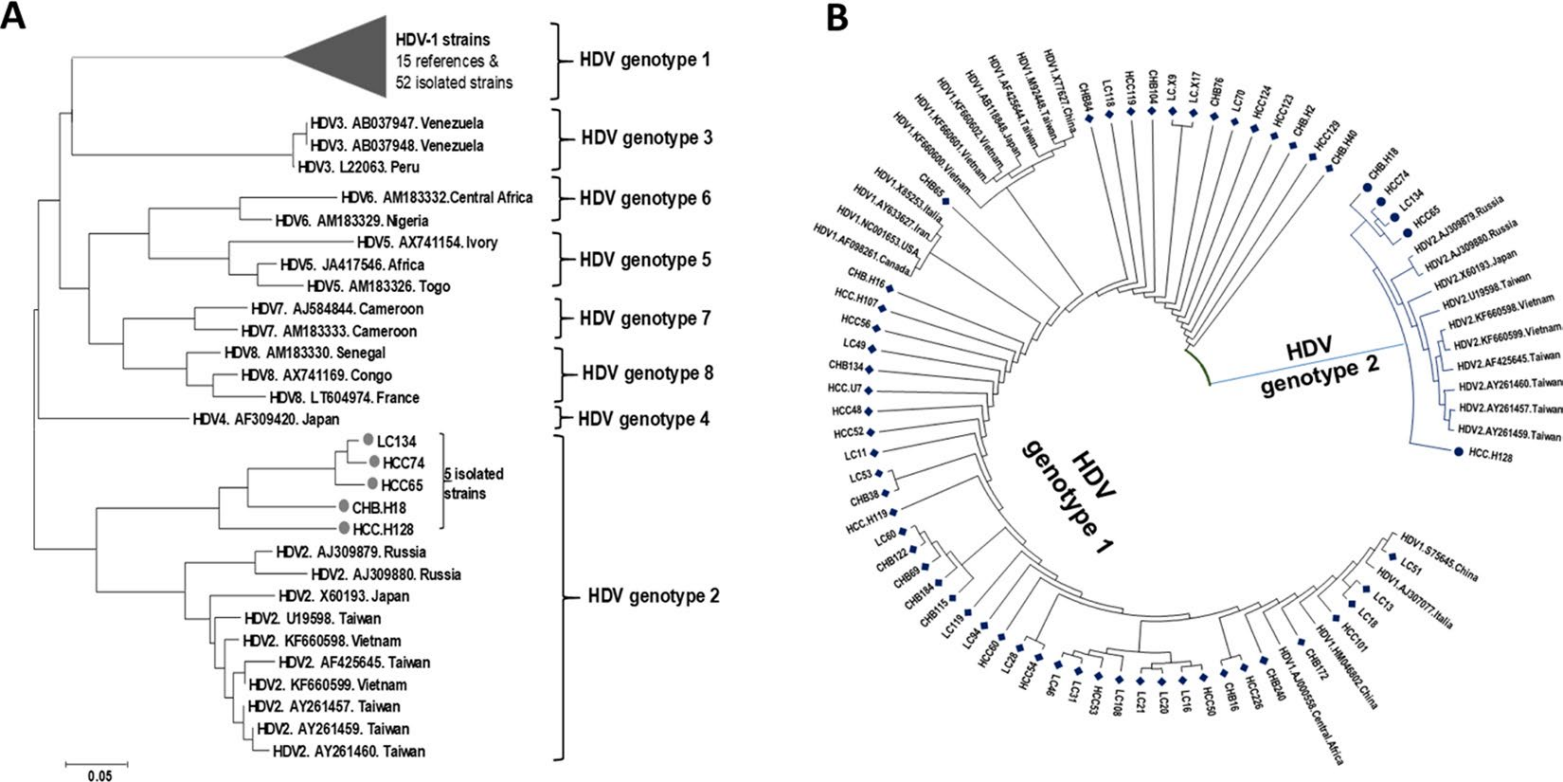
Phylogenetic analysis of isolated HDV genotypes. (**A**) A phylogenetic tree was constructed based on the alignment of 235 bp of 57 nucleotide sequences isolated from HDV/HBV co-infected patients. 39 full-length HDV genomes through HDV1-8 retrieved from NCBI database along with GenBank accession numbers were included for the analysis. A neighbor-joining tree was constructed with a bootstrap of 1000 replicates. The bar at the base of the tree indicates the scale for nucleotide substitutions per position. (**B**) The phylogenetic tree was constructed only for HDV genotype 1 and 2 sequences and involves 82 nucleotide sequences (25 references of full-length HDV genome retrieved from NCBI database, 52 strains of HDV genotype 1 (denoted as ♦) and 5 HDV genotype 2 (denoted as •) from HBV/HDV coinfected patients in our study group.

We then analyzed associations between HDV infection and liver function parameters in the HBV subgroups of CHB, LC and HCC. The results are given in Fig. 2 and Table 3. In the CHB group, there were no differences of all biochemical parameters between patients with and without HDV infection. In the LC group, AST and ALT levels were significantly higher in HDV/HBV coinfected patients than in HBV monoinfected patients (*P* < 0.05). In the HCC group, a similar trend applied to the total and direct bilirubin levels in comparison between HCC patients with and without HDV infection (median: 22 *vs*. 17 μmol/L, *P* = 0.017 and 9.8 *vs*. 5 μmol/L, *P* = 0.005, respectively). This was also seen when comparing patients with advanced liver disease (LC and/or HCC patients) with CHB patients (median: 35.5 vs. 19 μmol/L*, P* = 0.0014 and 12 vs. 6.7 μmol/L, *P* = 0.002, respectively).

**Table 3:** Association of HDV infection and clinical parameters in each HBV subgroup.

Groups	HDV status	HBV DNA (log_10_ copies/mL)	PLT (×10^3^/L)	AST (IU/L)	ALT (IU/L)	Total bilirubin (µmol/L)	Direct bilirubin (µmol/L)	Albumin (g/L)	Prothrombin (%)
CHB (n = 191)	HDV (−)	5.4 [2–10.3]	210 [65–416]	46 [14–6206]	60 [8–3390]	16.3 [5.5–551]	5.7 [1–349]	42 [9.8–51]	92 [35–267]
	HDV (+)	5.7 [2–8.9]	203 [95–262]	67 [26–883]	59 [26–1630]	17 [9.6–321]	7.35 [2.9–168]	42 [25–67]	98 [17–127]
LC (n = 147)	HDV (−)	5.4 [2–9]	89 [17.1–325]	74 [18–1221]	51 [8–1426]	30 [4.1–593]	10 [0.4–350]	31 [15–46]	57 [13–120]
	HDV (+)	3.9 [2–7.2]	91 [28–306]	98 [19–712]*	66 [14–1354]*	38 [9–358]	12 [2–238]	32 [26–47]	71.5 [29–100]
HCC (n = 208)	HDV (−)	4.6 [2–9.1]	166 [50–426]	58 [17–670]	49 [11–934]	17 [6–214]	5 [0.4–133]	38 [21–49]	85 [19–269]
	HDV (+)	4.5 [2–9.2]	166.5 [42–334]	56 [23–356]	38 [12–565]	21.7 [9.7–282]*	9.8 [1.3–189]**	36 [21–45]	78 [40–103]
HCC + LC (n = 355)	HDV (−)	4.67 [2–9.1]	128 [17.1–426]	63 [17–1221]	49 [8–1426]	19 [4.1–593]	6.7 [0.4–350]	36 [15–49]	77 [13–269]
	HDV (+)	4.3 [2–9.2]	104 [28–334]	78.5 [19–712]	55.5 [12–1354]	35.5 [9–358]**	12 [1.3–238]**	34 [21–47]	76 [29–103]

CHB: Chronic Hepatitis B; LC: Liver cirrhosis; HCC: Hepatocellular carcinoma; PLT: Platelet count; AST: Alanine aminotransferase; ALT: Aspartate aminotransferase. Values given are median and range; IU: international unit; *denotes *P* < 0.05; **denotes *P* < 0.005.

### HDV genotype distribution and its relation to clinical outcomes

Among the 71 HDV-RNA positive samples, 57 samples were successfully sequenced and genotyped. Phylogenetic analyses of the amplified fragment (235 bp) from HDV genomes of 57 sequences showed that HDV1 was the most frequent genotype (52/57, 91%); HDV2 was found in 5/57 (9%) only of HDV/HBV-coinfected patients (Fig. 2A,B). All sequences were submitted to the GenBank database (MG722912-MG722968).

We evaluated the impact of the HDV1 and HDV2 genotypes on the clinical outcome in HBV-infected patients by comparing the levels of biomedical parameters, including HBV-DNA loads, liver enzymes, bilirubin, albumin, prothrombin, and platelet counts. HBV-DNA levels were lower in HDV1-infected patients compared to HDV2-infected patients (not significant) (Fig. 1A). Comparisons of biochemical parameters were also not significant (data not shown).

## Discussion

In spite of effective HBV vaccines available, HDV/HBV coinfections are still of concern and underreported in many HBV-endemic regions. In Vietnam, although a universal hepatitis B vaccination program has been implemented since 2003 with a vaccine coverage was 97% in 2012 and a birth dose coverage increased to 75% in 2012 from 65% in 2006. The main route of transmission of hepatitis B in Vietnam is from mother to child. HBV infection still is one of the most serious public health problems with 10–15% of infected individuals in the general population and 20–40% among high risk groups such as drug users and HIV-positive individuals^19,24^. This suggests a high prevalence of HDV infection among Vietnamese HBsAg positive individuals. The minimum age of HBsAg positive patients was 12 years in our 2013 cohort, signifying that these individuals were not vaccinated by 2003. Furthermore, our study indicates that individuals at early adolescence are still at high risk, in a Vietnamese population that has a median age of 30 years. Therefore, in next decades, HBV and HBV/HDV coinfection may pose a significant health burden in Vietnam. Few data on the molecular epidemiology of HDV infection are available and the results are inconsistent regarding both the prevalence of infection and the distribution of HDV genotypes in Vietnam^20–23^. Here we describe a high HDV prevalence and confirm the predominance of the HDV1 genotype in northern Vietnam.

HDV infection rates vary considerably between countries in Africa, South America and parts of Asia^4,10,20,21,25^, while prevalences are low in northern Europe and North America, where HDV infections occur mostly among intravenous drug users^25^. A distinct geographic distribution of the HDV prevalence has been described for Vietnam. One study has shown that HDV infection rates varied in five regions across Vietnam, including Hanoi, Hai Phong, Da Nang, Khanh Hoa and Can Tho^21^. HDV seroprevalences were high in northern Vietnam (30.2% and 29.4% in Hanoi and Hai Phong, respectively), but lower in southern Vietnam (8.1% in Khanh Hoa and 12.5% in Can Tho) and in Central Vietnam (5.3% in Da Nang). A previous study conducted in a cohort of HBV-infected patients collected over a decade ago had already shown a high HDV-RNA prevalence in northern Vietnam (15.4%)^23^. The prevalence of HDV-RNA was 10% in chronic hepatitis B carriers collected in 2015 in Central Vietnam^22^. The difference in HDV-RNA prevalences between these two studies reflects distinct geographic distributions of the infection. Our findings of a high HDV infection rate in northern Vietnam (13%) corroborate the earlier results^21,23^ and indicate that HDV infection rates did not decline during the last decade in northern Vietnam, although the immunization coverage against HBV is well established. However, it is early enough to see a potential influence of HBV vaccination on the burden of HDV infection after only 10–12 years of vaccination campaign by which the target population of the vaccination program represents for/by newborns and infants.

Varying distributions of HDV genotypes in North and South Vietnam have recently been reported^21–23^, with the HDV1 genotype being predominant in all parts of Vietnam^21^. In contrast, HDV1 has been shown to prevail in northern Vietnam with 90%^23^, but accounted for only 20% in central Vietnam^22^. We here confirm the predominance of HDV1 (HDV1, 91% *vs*. HDV2, 9%) in northern Vietnam, consistent with the finding that HDV1 is worldwide the most common genotype, while the other genotypes are rather restricted to distinct geographical regions^8,11,26,27^.

Concomitant HDV and HBV infections significantly increase the risk of HCC development and liver decompensation at an early stage of coinfection^8,10,28,29^. In our study, HDV positivity rates were particularly high in the LC group, followed by the HCC and CHB groups. Patients with Child-Pugh scores B and C had higher infection rates compared to patients scored as Child-pugh A, indicating that HDV infection is in fact associated with progression to liver cirrhosis. The levels of liver enzymes and bilirubin in patients with advanced liver diseases (LC, HCC) in HDV/HBV-coinfected patients were elevated compared to HDV-negative patients. Like HBV/HCV coinfection, HBV/HDV coinfection is associated with diverse patterns of reciprocal inhibition of viral replication^30^. Although the difference was not significant, our data show that HBV-DNA loads were lower in HDV/HBV coinfection compared to HBV monoinfection, in part supporting the previous finding that HDV-infection suppresses HBV replication^31,32^. While other studies have demonstrated an impact of HDV genotypes on the clinical presentation of HBV-infected patients^3,10,22^, our study did not show any significant association of HDV genotypes with liver function test, HBV-DNA loads, and AFP levels. This lack of association may result from the small number of patients infected with HDV2.

In conclusion and in comparison with the situation a decade ago, HDV infection is still a serious medical problem in Vietnam. HDV-RNA positivity in HBV infected patients is high and HDV1 is the predominant genotype circulating in northern Vietnam. A continuing high prevalence of HDV infection in hepatitis B patients increases the disease burden. The National authorities in Vietnam should reconsider the operational clinical protocols to include HDV nucleic acid testing to minimize the associated risk of liver cirrhosis and hepatocellular carcinoma in patients infected with chronic HBV.

## Subjects and Methods

### Clinical patients

Five hundred forty-six Vietnamese HBV-infected patients were consecutively enrolled at the 108 Military Central Hospital, Hanoi, Vietnam, between 2013 and 2015. All participants were negative for anti-HCV and anti-HIV antibodies as assessed by routine ELISA assays. None of the study participants had a history of alcohol or drug abuse. All LC and HCC patients and most CHB patients were admitted to hospital for specific treatment. HBV-DNA loads and biochemical liver function parameters, including the levels of alanine transaminase (ALT), aspartate transaminase (AST), total bilirubin and direct bilirubin as well as albumin and prothrombin were assessed. Patients were classified into the clinical subgroups of chronic hepatitis B (CHB, n = 191), liver cirrhosis (LC, n = 147), and hepatocellular carcinoma (HCC, n = 208). The diagnostic criteria applying to each subgroup have previously been described^33^. The liver function of LC and HCC patients were categorized according to Child-Pugh scores (Child-A, B, and C)^34^. Staging of HBV-related HCC was assessed according to the Barcelona Clinic Liver Cancer (BCLC) strategy^35^. Blood sampling of all patients were performed on admission. Whole blood and serum samples were stored at −80 °C until use.

### Ethics statement

Informed written consent was obtained after detailed explanation of the study at the time of blood and serum sampling from all participants or from their parents if subjects were <18 years old. The study was approved by the institutional Review Board of the 108 Military Central Hospital, Hanoi, Vietnam. All experiments were performed in accordance with relevant guidelines and regulations.

### Nucleic acid extraction and cDNA synthesis

Viral RNA was isolated from serum obtained from the study participants (QIAamp Viral RNA Mini Kit; Qiagen GmbH, Hilden, Germany). HDV-RNA was reverse transcribed into cDNA using the High-Capacity cDNA Reverse Transcription Kit (Thermo Fisher Scientific, Foster City, CA, USA) following the manufacturer’s instructions.

### HDV-RNA detection

Nested PCR assays specific for the identification of HDV were performed using four highly conserved primer pairs (Table 4), representing all eight currently recognized HDV genotypes as previously described^22^. PCR amplification was carried out in 25 μl reaction volumes (5 ng cDNA, 10x buffer [20 nM Tris-HCl, 50 nM KCl, 2 nM MgCl_2_], 0.2 nM dNTPs, 0.4 nM MgCl_2,_ 0.6 μM specific primer pairs, and 1 unit Taq polymerase). The first PCR round was performed with primer pairs HDV57_F and HDV60_R (nucleotides 299 to 770, according to NC001653). PCR conditions included an initial denaturation step (94 °C, 4 min), 32 cycles of 30 sec at 94 °C denaturation, 30 sec at 54 °C annealing, 30 sec at 72 °C extension, followed by a final extension at 72 °C for 10 min. The second PCR round was performed using the primer pairs HDV48_F and HDV54_R to amplify the inner fragment (nucleotides 313 to 485, according to NC001653). PCR conditions included initial denaturation for 4 min at 94 °C, followed by 35 cycles (denaturing at 94 °C for 30 sec, annealing at 58 °C for 30 sec and extension at 72 °C for 30 sec), with a final 10 min extension step at 72 °C. Amplicons obtained in the second PCR round were visualized on 1.5% agarose gels. Samples were considered positive when HDV-RNA was detected at least twice in three consecutive PCR runs.

**Table 4:** Primers used for HDV detection.

Primers	Sequence (5′-3′)	Position	PCR round
HDV04_F	GGATGCCCAGGTCGGACCG	856–874	1^st^ round PCR
HDV05_R	AAGAAGAGRAGCCGGCCCGY	1159–1179	1^st^ round PCR
HDV06_F	ATGCCATGCCGACCCGAAGA	888–907	2^nd^ round PCR
HDV07_R	GGGGAGCGCCCGGDGGCGG	1104–1122	2^nd^ round PCR
HDV57_F	GAGAAMYCACCTCCAGAGGA	299–318	1^st^ round PCR
HDV60_R	TCCCATTCGCCATTACCGA	752–770	1^st^ round PCR
HDV48_F	AGAGGACCCCTTCAGCGAAC	313–332	2^nd^ round PCR
HDV54_R	CCGGGATAAGCCTCACTCG	467–485	2^nd^ round PCR

### HDV genotyping and phylogenetic analysis

In order to define HDV genotypes, nested PCRs were performed using primer pairs HDV04_F and HDV05_R for the first round for amplification of the outer fragment (nucleotides 856 to 1179, according to NC001653; Table 4). For the second round we used primer pairs HDV06_F and HDV07_R to amplify the inner fragment (nucleotides 888 to 1122, according to NC001653). Cycling parameters were identical with those applied for HDV detection as described above. The inner PCR amplicons obtained in the second round were purified using either the GFX PCR DNA and Gel Band Purification Kit (GE Healthcare Europe GmbH, Freiburg, Germany) or the Exo-SAP-IT kit (USB, Affymetrix, MA, USA) following the manufacturer’s instructions. Purified PCR products were subsequently used as sequencing templates using the ABI 3130XL system and the BigDye terminator v.1.1 sequencing kit (Applied Biosystems, Foster City, CA, USA).

Phylogenetic trees were constructed using the MEGA7 software (www.megasoftware.net). References of the full genomes of eight HDV genotypes (HDV1-8) were retrieved from available NCBI GenBank data, including the sequences of genotypes HDV1 (AB118848, AF098261, AF425644, AJ000558, AJ307077, AY633627, HM046802, KF660600, KF660601, KF660602, M92448, NC001653, S75645, X77627, X85253), HDV2 (KF660598, AF425645, AJ309879, AJ309880, AY261457, AY261459, AY261460, KF660599, U19598, X60193), HDV3 (AB037947, AB037948, L22063), HDV4 (AF309420, HM309420), HDV5 (AM183326, AX741154, JA417546), HDV-6 (AM183329, AM183332), HDV7 (AJ584844, AM183333) and HDV8 (AM183330, AX741169, LT604974). All sequences were aligned using BioEdit software version 7 (http://www.mbio.ncsu.edu/BioEdit/page2.html) and the CLUSTAL Muscle algorithm^36^. Phylogenetic trees were constructed using the neighbour joining method^37^ and the Kimura-2 model^38^. Statistical robustness and reliability of the branching order was confirmed by bootstrap analysis using 1000 iterations.

### Statistical analysis

For statistical analyses the R software (https://www.r-project.org) and GraphPad Prism 7 (http://www.graphpad.com) were applied. Chi-square or Fisher’s exact test were performed to test for differences of categorical variables between two or more than two groups. Wilcoxon-Mann-Whitney and Kruskal-Wallis tests were used to compare nonparametric data of quantitative variables between two or more than two groups. The level of significance was *P* < 0.05.
